# Tyrannosaurs as long-lived species

**DOI:** 10.1038/srep19554

**Published:** 2016-01-21

**Authors:** Byung Mook Weon

**Affiliations:** 1 Soft Matter Physics Laboratory, School of Advanced Materials Science and Engineering, SKKU Advanced Institute of Nanotechnology (SAINT), Sungkyunkwan University, Suwon 440-746, Korea

## Abstract

Biodemographic analysis would be essential to understand population ecology and aging of tyrannosaurs. Here we address a methodology that quantifies tyrannosaur survival and mortality curves by utilizing modified stretched exponential survival functions. Our analysis clearly shows that mortality patterns for tyrannosaurs are seemingly analogous to those for 18th-century humans. This result suggests that tyrannosaurs would live long to undergo aging before maximum lifespans, while their longevity strategy is more alike to big birds rather than 18th-century humans.

Tyrannosaurs including *Tyrannosaurus rex* (shortly *T. rex* meaning *tyrant lizard king*) are very popular to the public as well as among paleontologists although they became extinct 66 million years ago on the Earth^1^. Many mysteries about population ecology and actual behavior of tyrannosaurs have been resolved thanks to modern technologies and collective data in paleobiology^2^,^3^,^4^. In particular, rigorous anatomic methods have been developed^5^ and eventually reliable life tables for tyrannosaurs were estimated^6^ (a few data were updated later^7^). Using their demographic data, tyrannosaur aging dynamics was carefully interpreted^8^. Gompertz function^9^ or Weibull function^10^ was utilized to quantify tyrannosaur survival curves^6^,^8^, but both might be insufficient to appropriately describe complicated biological survival curves. Suitable mathematical descriptions and statistical methods are still required to quantify survival and mortality curves of tyrannosaurs^11^.

Here we address a methodology that enables us to appropriately quantify tyrannosaur survival and mortality curves by utilizing modified stretched exponential survival functions, which we have developed to precisely quantify human demographics^12^,^13^,^14^,^15^. We find a demographic analogy between tyrannosaurs and 18th-century humans despite scale and ecological differences. Interestingly, mortality patterns for tyrannosaurs resemble those for 18th-century humans: probably tyrannosaurs would be able to live so long to undergo aging before maximum lifespans, while their longevity strategy would be more alike to big birds rather than 18th-century humans. We attribute longevity of tyrannosaurs to late sexual maturity, large body size, and rapid growth rate, which would be favorable for longevity.

## Results

We adopt the demographic data for tyrannosaurs adapted from the survival curves for *Albertosaurus sarcophagus*, *Tyrannosaurus rex*, and *Gorgosaurus libratus*^6^ (the data for *Albertosaurus sarcophagus* were taken from the updated data^7^). Tyrannosaurs are known to have high neonate mortality before age 2 years: hypothesized neonate mortality is 60%^6^,^7^. Therefore we include 60% neonate mortality into the estimated proportion of individuals alive at the beginning of each year class *l*_*x*_ in the tyrannosaur life tables^6^,^7^ to become *l*_*x*_ = 1.0 at age 0 by taking 0.4*l*_*x*_ (see Supplementary Data). Previous demographic analysis for tyrannosaurs might be imperfect with Gompertz and Weibull functions because of no correction for 60% neonate mortality^6^,^8^.

To appropriately quantify tyrannosaur survival curves that have 60% neonate mortality, we utilize modified stretched exponential survival functions, described as *s*(*x*) = exp(−(*x*/*α*)^*β*(*x*)^). In statistics, the mortality rate (equally the hazard function or the force of mortality), *μ*(*x*) = *d*ln(*s*(*x*))/*dx*, is mathematically linked to the survival rate, *s*(*x*), which monotonically declines from 1 to 0 as age *x* increases. Here, the stretched exponent *β* is given by *β*(*x*) = ln[−ln(*s*(*x*))]/ln(*x*/*α*) as a function of age, and the characteristic life *α* is accurately measured by detecting the interception point between *s*(*x*) and *s*(*α*) = exp(−1). The rough *α* estimate could be obtained from a linear regression from two data points that exist just above (+1p) and just below (−1p) the exp(−1) point for survival data (taken from tyrannosaur life tables^6^,^7^) that are too sparse to produce the exact *α* estimate, as illustrated in Fig. S1. The *α* value can serve as a good alternative to the life expectancy at birth^15^,^16^. The age dependence of the stretched exponent is the critical difference between the modified stretched exponential^12^,^13^,^14^,^15^ and the classical stretched exponential (known as the Kohlrausch-Williams-Watts^17^,^18^ or Weibull^10^) functions. Mathematically, the stretched exponential function is a superposition of exponential functions^15^ and the age-dependent stretched exponent reflects the population heterogeneity^19^,^20^. The assessment of *α* and the determination of *β*(*x*) for a survival curve enables the determination of an exact formula for the mortality curve through *μ*(*x*) = (*x*/*α*)^*β*(*x*)^[*β*(*x*)/*x* + ln(*x*/*α*)*dβ*(*x*)/*dx*]^12^. Empirically, a polynomial formula of *β*(*x*) describes the *β*(*x*) patterns quite well^12^,^13^,^14^,^15^. This methodology has been well accepted in the recent literature^21^,^22^.

**Figure 1:**
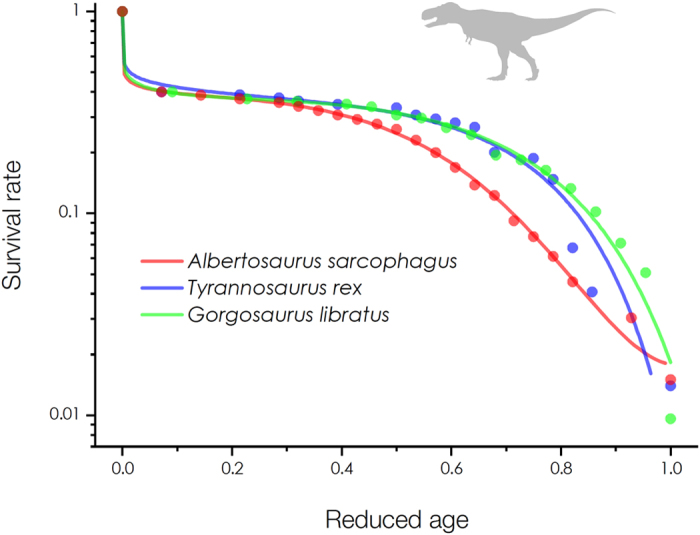
Survival curves of tyrannosaurs. The demographic data for tyrannosaurs were adapted from the survival curves by calibrating the 60% neonate mortality^6^,^7^ (the dots). The data were fit by the modified stretched exponential functions (the solid lines) as *s*(*x*) = exp(−(*x*/*α*)^*β*(*x*)^) by measuring the characteristic life *α* and the stretched exponent *β*(*x*). The reduced age *x*/*ω* is taken by dividing the tyrannosaur age *x* by their maximum lifespan *ω*.

**Figure 2:**
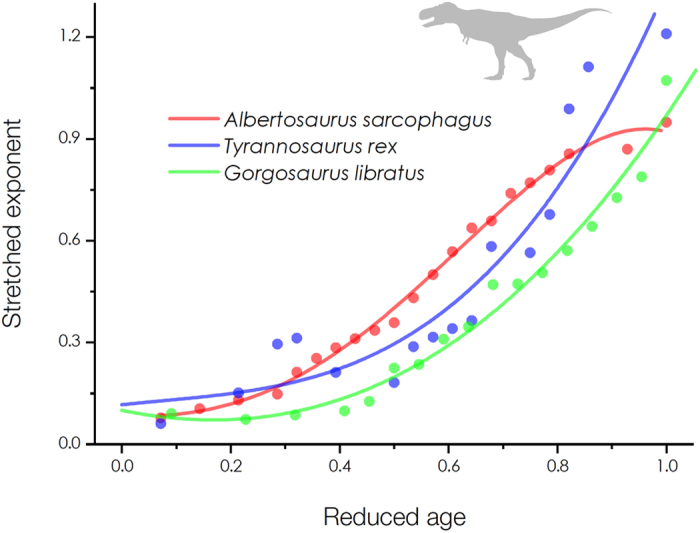
Age-dependent patterns of stretched exponents for tyrannosaurs. The age-dependent stretched exponents *β*(*x*) for each survival curve were formulated by polynomial functions (the solid lines).

**Figure 3:**
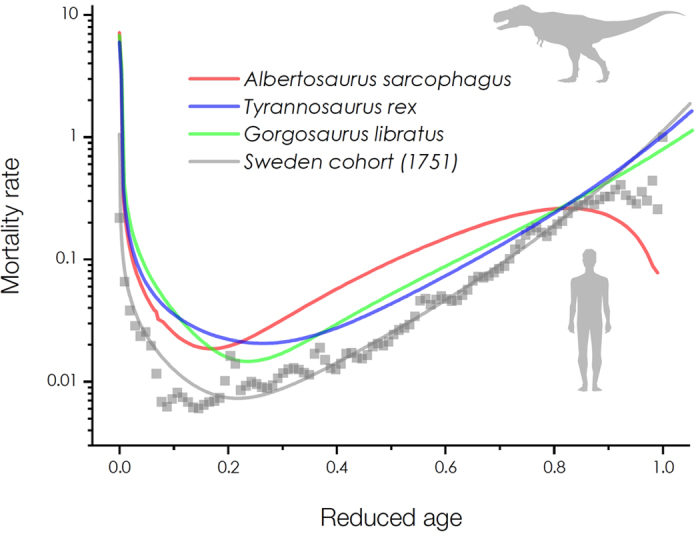
Mortality curves of tyrannosaurs and 18th-century humans. Suitable descriptions of mortality curves were obtained from *s*(*x*) (Fig. 1) and *β*(*x*) (Fig. 2). We compared mortality patterns for tyrannosaurs (the solid lines) and 18th-century humans (the closed squares for the 1751 Swedish cohorts of males and females; the lowest solid line for the modeled mortality curve).

Here, we present representative results for successful quantifications of the survival curves for tyrannosaurs with 60% neonate mortality as illustrated in Fig. 1. The survival data of tyrannosaurs are depicted as the dots and the mathematical fits by our methodology are depicted as the solid lines. For ecological comparison, the tyrannosaur age is normalized by the reduced age *x*/*ω* (by dividing the age *x* by the maximum lifespan *ω* ≈ 28 years for *Albertosaurus sarcophagus* and *Tyrannosaurus rex* and *ω* ≈ 22 years for *Gorgosaurus libratus*, as suggested^6^). These results were obtained by fitting modified stretched exponential functions *s*(*x*) = exp(−(*x*/*α*)^*β*(*x*)^). The characteristic lives *α* for *Albertosaurus sarcophagus*, *Tyrannosaurus rex*, and *Gorgosaurus libratus* were measured by regression analyses as *α* = 6.168, 8.433, and 5.251 years, respectively. The stretched exponents *β*(*x*) = ln[−ln(*s*(*x*))]/ln(*x*/*α*) for each survival curve were converted in Fig. 2 (as depicted as the dots). The apparent age-dependent *β*(*x*) patterns were formulated by polynomial functions (as depicted as the solid lines) as: *β*(*x*) = 0.08141–5.63062 × 10^−4^ *x* + 7.13967 × 10^−4^ *x*^2^ + 1.23348 × 10^−4^ *x*^3^ − 3.9252 × 10^−6^ *x*^4^ (adj. *R*^2^ = 0.99262) for *Albertosaurus sarcophagus*, *β*(*x*) = 0.11618 + 0.00616*x* − 3.96359 × 10^−4^ *x*^2^ + 6.22384 × 10^−5^ *x*^3^ (adj. *R*^2^ = 0.87897) for *Tyrannosaurus rex*, and *β*(*x*) = 0.09969 − 0.01496*x* + 0.00187*x*^2^ + 2.74776 × 10^−5^*x*^3^ (adj. *R*^2^ = 0.97026) for *Gorgosaurus libratus*. Finally suitable descriptions of tyrannosaur mortality curves were obtained from *s*(*x*) using their relation *μ*(*x*) = *d*ln(*s*(*x*))/*dx* as depicted in Fig. 3. The neonate mortality for ages 0–2 years were modeled by extrapolating *β*(*x*) in Fig. 2. These mortality patterns, particularly exhibiting high neonate mortality, are consistent with previous interpretation for tyrannosaur mortality patterns^6^,^7^. Interestingly, the exponential growth in mortality rates for adult tyrannosaurs (around the reduced ages *x*/*ω* ~ 0.6 in Fig. 3) seems to follow the Gompertz mortality law^9^, as important features in human aging dynamics^14^.

To demographers, tyrannosaur survival curves look similar to survival curves for humans lived in the past centuries^14^. Tyrannosaurs are believed to suffer relatively high initial mortality but low juvenile mortality, and have an accelerated aging pattern similar to that of humans and large mammals^6^,^8^. To clarify this similarity, we selected demographic data for Swedish cohorts born in the year 1751 that are the most reliable data taken from the Human Mortality Database (www.mortality.org, accessed on 9 March 2015). These data would be representative for 18th-century humans: huge demographic data and analyses for humans were quite well documented in the literature^14^.

For the Swedish 1751 cohorts, we applied the same demographic analysis with *α* = 52.3256 years, *ω* = 103 years, and *β*(*x*) = 0.35852 + 0.00425*x* + 2.90294 × 10^−4^*x*^2^ (adj. *R*^2^ = 0.99169) and finally obtained a representative human mortality pattern in Fig. 3 (the solid grey line). The 18th-century human survival data are similar to the tyrannosaur data but the modern Swedish mortality curves tend to shift downward as the calendar year increases^14^. This comparative analysis clearly shows that mortality patterns for tyrannosaurs are closely akin to those for 18th-century humans, implying that tyrannosaurs would live so long to undergo aging before their maximum lifespans^6^.

## Discussion

Our result implies that mortality patterns of tyrannosaurs are seemingly similar to those of 18th-century humans as demonstrated in Fig. 3, but this similarity may be a mathematical coincidence. Tyrannosaurs are more closely related to birds than mammals^8^, while interestingly their mortality patterns resemble those for 18th-century humans. Our analysis in mortality curves with our mothodology suggests that tyrannosaurs would undergo aging before reaching their maximum lifespans, which seems analogous to human aging in adulthood (for the reduced ages *x*/*ω* ~ 0.4–1.0).

However, there is a big difference between tyrannosaurs and 18th-century humans in mortality patterns. *Tyrannosaurus* was reproductively mature by 18 years^23^ and lived up to 28 years maximally^6^, indicating the relatively late sexual maturity at the reduced ages *x*/*ω* ~ 0.6. For tyrannosaurs, the gradual increase in mortality rates start at the reduced ages *x*/*ω* ~ 0.2, while for humans, the mortality rates exponentially increase after the reproductive ages (*x*/*ω* ~ 0.2) indicating aging in adulthood. This analysis suggests that tyrannosaurs would undergo aging before maximum lifespans for the reduced ages *x*/*ω* ~ 0.6–1.0, while their longevity strategy would be quite different with humans.

**Figure 4:**
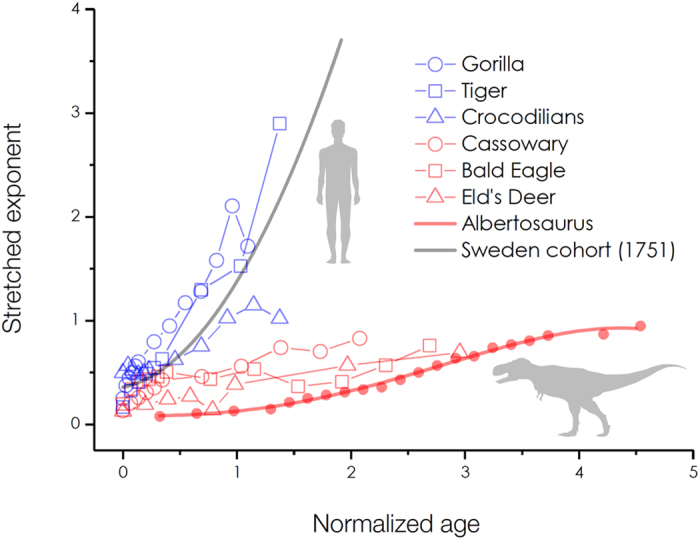
Species-dependent sensitivity of stretched exponents. The age-dependent stretched exponents *β*(*x*) with respect to the normalized age *x*/*α* for various species^24^ exhibit a significant difference between 18th-century humans and tyrannosaurs. The species-dependent scale effect can be ruled out by using the normalized age *x*/*α*^14^. Assessing the *β*(*x*) patterns is useful in understanding longevity strategy across species.

Species-dependent sensitivity of our methodology was demonstrated for flies, worms, humans, and tyrannosaurs^13^. Our methodology is quite sensitive to the shape and the scale of any survival curves^14^,^15^. Specifically, *living longer* is associated with the scale variance of a survival curve, whereas *growing older* is associated with the shape variance^14^. The scale effect can be ruled out by normalizing the age with the characteristic life as *x*/*α*, enabling us to compare the shape effect (the stretched exponent) of a survival curve. On this basis, we can compare the shape variance of survival curves for selected species such as gorilla, tiger, crocodilians, cassowary, bald eagle, and deer, as demonstrated in Fig. 4, by taking demographic data from the literature^24^. Our methodology is useful in characterizing the survival curves that vary with species.

Analyzing the stretched exponents would help evaluation of longevity strategy across species. Although survival and mortality curves [Figs 1 and 3] look very similar between tyrannosaurs and 18th-century humans, their stretched exponent patterns [Fig. 4] are significantly different. The stretched exponents *β*(*x*) with respect to the normalized age *x*/*α* in Fig. 4 show a clear difference in longevity strategy between 18th-century humans and tyrannosaurs. For 18th-century humans (Sweden cohort in 1751, *α* = 52.326 years), the *β*(*x*) curves are similar to those of apes (gorilla, *α* = 36.485 years), carnivores (tigers, *α* = 14.529 years), or crocodilians (*α* = 21.785 years), whereas those of *Albertosaurus sarcophagus* (*α* = 6.168 years) show similar patterns with hoofstock (deer, *α* = 5.076 years), ratites (cassoway, *α* = 14.437 years), or raptors (bald eagle, *α* = 13.009 years). This analysis suggests that tyrannosaurs would live longer than other species in terms of the normalized age.

Tyrannosaurs would exhibit late sexual maturity, large body size, and rapid growth rate, which would be favorable for longevity. First, the late sexual maturity might be a good feature of long lifespan^8^. The difference between crocodilians and tyrannosaurs would be attributed to the maturity rate difference^23^, which is reflected in the *β*(*x*) patterns. The *β*(*x*) pattern by relatively early maturity for crocodilians is alike to that for humans, while the *β*(*x*) pattern by relatively late maturity is found for tyrannosaurs alike to big birds such as ratites and raptors. For tyrannosaurs, the reproductive age ~18 years^23^ is later than the characteristic life ~6 years, while the reproductive age for humans is earlier than the characteristic life.

Second, larger animals usually live longer: there is an allometric scaling law between the body size and the lifespan^25^,^26^. For most mammals, the lifespan (*τ*) increases with the body mass (*m*) as a scaling of *τ* ~ *m*^1/4^^25^,^26^. Humans are exceptional in such allometric laws: humans have evolved to take large brains, which would be favorable to acquire exceptional longevity^27^,^28^. Typical mammals have 17.5 years on average as the maximum lifespan, while humans can live up to 125 years^12^,^15^,^29^. For tyrannosaurs, the maximum lifespan (~28 years^6^) would be longer than for most birds (15.6 years on average^29^) or reptiles (11.2 years on average^29^). Presumably, the large body size of tyrannosaurs would be favorable for their longevity^4^.

Finally, there would be benefits from predation relief by rapid growth^2^,^4^ for longevity of tyrannosaurs. Probably becoming giants through rapid growth^4^ or becoming apex predators^3^ would be favorable to acquire exceptional benefits for releases from predation in early life, which would be good for longevity, regardless of uncertainty on whether they were primarily predators or scavengers^1^,^2^.

Our methodology would be applicable to paleodemographic studies, which would be useful for quantitative analyses on dinosaur survival curves and aging dynamics. Fitting a proper growth curve^30^,^31^ and taking a large sample size^11^ are essential for constructing a reliable life table. Further studies are required to build more reliable life tables and to derive adequate statistical methodologies in quantifying aging dynamics for tyrannosaurs. Our methodology is a descriptive method to quantify complexity in aging dynamics^15^ and other approaches would be complementary for more understanding of tyrannosaur paleobiology and aging dynamics^8^,^32^,^33^. Comparative analyses of aging for large animals including dinosaurs, mammals, and birds would help identify aging mechanisms across species^8^,^32^.

In summary, we suggest a methodology that enables us to appropriately quantify tyrannosaur survival and mortality curves by utilizing modified stretched exponential survival functions. This analysis leads to a conceivable conclusion that mortality patterns for tyrannosaurs resemble those for humans, while longevity strategy for tyrannosaurs is more alike to big birds rather than humans. Probably tyrannosaurs would be able to live so long to undergo aging before their maximum lifespans, which would be plausible by benefits from late sexual maturity, large body size, and rapid growth rate.

## Additional Information

**How to cite this article**: Weon, B. M. Tyrannosaurs as long-lived species. *Sci. Rep*. **6**, 19554; doi: 10.1038/srep19554 (2016).

## Supplementary Material

Supplementary Information

## References

[b1] Erickson G. M. Breathing life into *T. rex*. Sci. Am. 23, 38–45 (2014).

[b2] Brusatte S. L. . Tyrannosaur paleobiology: new research on acent exemplar organisms. Science 329, 1481–1485 (2010). 20847260 10.1126/science.1193304

[b3] DePalma II R. A., Burnham D. A., Mattin L. D., Rothschild B. M. & Larson P. L. Physical evidence of predatory behavior in *Tyrannosaurus rex*. Proc. Natl. Acad. Sci. USA 110, 12560–12564 (2013). 23858435 10.1073/pnas.1216534110 PMC3732924

[b4] Erickson G. M. On dinosaur growth. Annu. Rev. Earth Planet. Sci. 42, 675–697 (2014).

[b5] Erickson G. M. . Gigantism and comparative life-history parameters of tyrannosaurid dinosaurs. Nature 430, 772–775 (2004). 15306807 10.1038/nature02699

[b6] Erickson G. M., Currie P. J., Inouye B. D. & Winn A. A. Tyrannosaur life tables: an example of nonavian dinosaur population biology. Science 313, 213–217 (2006). 16840697 10.1126/science.1125721

[b7] Erickson G. M., Currie P. J., Inouye B. D. & Winn A. A. A revised life table and survivorship curve for *Albertosaurus sarcophagus* based on the Dry Island mass death assemblage. Can. J. Earth Sci. 47, 1269–1275 (2010).

[b8] Ricklefs R. E. Tyrannosaur ageing. Biol. Lett. 3, 214–217 (2007). 17284406 10.1098/rsbl.2006.0597 PMC2375931

[b9] Gompertz B. On the nature of the function expressive of the law of human mortality. Philos. Trans. R. Soc. Lond. A 115, 513–580 (1825).

[b10] Weibull W. A. A statistical distribution function of wide applicability. J. Appl. Mech. 18, 293–1951 (1951).

[b11] Stelnsaltz D. & Orzack S. H. Statistical methods for paleodemography on fossil assemblages having small numbers of specimens: an investigation of dinosaur survival rates. Paleobiology 37, 113–125 (2011).

[b12] Weon B. M. & Je J. H. Theoretical estimation of maximum human lifespan. Biogerontology 10, 65–71 (2009). 18560989 10.1007/s10522-008-9156-4

[b13] Weon B. M. & Je J. H. Plasticity and rectangularity in survival curves. Sci. Rep. 1, 104 (2011). 22355622 10.1038/srep00104 PMC3216589

[b14] Weon B. M. & Je J. H. Trends in scale and shape of survival curves. Sci. Rep. 2, 504 (2012). 22792436 10.1038/srep00504 PMC3394084

[b15] Weon B. M. A solution to debates over the behavior of mortality at old ages. Biogerontology 16, 375–381 (2015). 25650286 10.1007/s10522-015-9555-2

[b16] Wrycza T. F. & Baudisch A. The pace of aging: intrinsic time scales in demography. Demogr. Res. 30, 1571–1590 (2014).

[b17] Kohlrausch R. Theorie des elektrischen rckstandes in der leidener flasche. Pogg. Ann. Phys. Chem. 91, 179–214 (1854).

[b18] Williams G. & Watts D. C. Non-symmetrical dielectric relaxation behavior arising from a simple empirical decay function. Trans. Faraday Soc. 66, 80–85 (1970).

[b19] Carnes B. A. & Olshansky S. J. Heterogeneity and its biodemographic implications for longevity and mortality. Exp. Gerontol. 36, 419–430 (2001). 11250115 10.1016/s0531-5565(00)00254-0

[b20] Avraam D., de Magalhaes J. P. & Vasiev B. A mathematical model of mortality dynamics across the lifespan combining heterogeneity and stochastic effects. Exp. Gerontol. 48, 801–811 (2013). 23707231 10.1016/j.exger.2013.05.054

[b21] Amorim M., Ferreira S. & Couto A. A conceptual algorithm to link police and hospital records based on occurrence of values. Transp. Res. Procedia 3, 224–233 (2014).

[b22] Holmes T. H. & Lewis D. B. Bayesian immunological model development from the literature: example investigation of recent thymic emigrants. J. Immunol. Methods 414, 32–50 (2014). 25179832 10.1016/j.jim.2014.08.001 PMC4259859

[b23] Lee A. H. & Werning S. Sexual maturity in growing dinosaurs does not fit reptilian growth models. Proc. Natl. Acad. Sci. USA 105, 582–587 (2008). 18195356 10.1073/pnas.0708903105 PMC2206579

[b24] Kohler I. V., Preston S. H. & Lackey L. B. Comparative mortality levels among selected species of captive animals. Demogr. Res. 15, 413–434 (2006).

[b25] West G. B., Brown J. H. & Enquist B. J. A general model for the origin of allometric scaling laws in biology. Science 276, 122–126 (1997). 9082983 10.1126/science.276.5309.122

[b26] Bejan A. Why the bigger live longer and travel farther: animal, vehicles, rivers and the winds. Sci. Rep. 2, 594 (2012). 22924107 10.1038/srep00594 PMC3426796

[b27] Gonzalez-Lagos C., Sol D. & Reader S. M. Large-brained mammals live longer. J. Evol. Biol. 23, 1064–1074 (2010). 20345813 10.1111/j.1420-9101.2010.01976.x

[b28] Hofman M. A. Evolution of the human brain: why bigger is better. Front. Neuroanat. 8, 15 (2014). 24723857 10.3389/fnana.2014.00015 PMC3973910

[b29] Hulbert A. J., Pamplona R., Buffenstein R. & Buttemer W. A. Life and death: metabolic rate, membrane composition, and life span of animals. Physiol. Rev. 87, 1175–1213 (2007). 17928583 10.1152/physrev.00047.2006

[b30] Myhrvold N. P. Revisiting the estimation of dinosaur growth rates. PLoS ONE 8, e81917 (2013). 24358133 10.1371/journal.pone.0081917 PMC3864909

[b31] Myhrvold N. P. Problems in Erickson *et al*. 2009. Anat. Rec. 298, 489-493 (2015). 10.1002/ar.23101 PMC4350431 25403556

[b32] Ricklefs R. E. Insights from comparative analyses of aging in birds and mammals. Aging Cell 9, 273–284 (2010). 20041859 10.1111/j.1474-9726.2009.00542.x PMC2858791

[b33] Wrycza T. F., Missov T. I. & Baudisch A. Quantifying the shape of aging. PLoS ONE 10, e0119163 (2015). 25803427 10.1371/journal.pone.0119163 PMC4372288

