# Supplementary material for: Tyrannosaurs as long-lived species
Source: Sci Rep. 2016 Jan 21;6:19554. doi: 10.1038/srep19554 (PMC4726238; doi:10.1038/srep19554)

Tyrannosaurs as long-lived species

Byung Mook Weon*

Soft Matter Physics Laboratory, School of Advanced Materials Science and Engineering,

SKKU Advanced Institute of Nanotechnology (SAINT),

Sungkyunkwan University, Suwon 440-746, Korea

**E-mail: bmweon@skku.edu*

Supplementary Data and Figure S1 are included.

Supplementary Data

Erickson et al.'s (2006, 2010) survivorship data for tyrannosaurs were modified to be l_x = 1.0 at age 0 by including hypothesized neonate mortality of 60%.

	Erickson et al. (2010)			Erickson et al. (2006)			Erickson et al. (2006)	
year	*Albertosaurus sarcophagus*	calibrated with l_2	year	*Tyrannosaurus rex*	calibrated with l_2	year	*Gorgosaurus libratus*	calibrated with l_2
2	l_2 = 1.0000	0.4000	2	l_2 = 1.0000	0.4000	2	l_2 = 1.0000	0.4000
4	0.9615	0.3846	6	0.9670	0.3868	5	0.9230	0.3692
6	0.9230	0.3692	8	0.9340	0.3736	7	0.8970	0.3588
8	0.8845	0.3538	9	0.9010	0.3604	9	0.8710	0.3484
9	0.8460	0.3384	11	0.8680	0.3472	10	0.8450	0.3380
10	0.8075	0.3230	14	0.8350	0.3340	11	0.7680	0.3072
11	0.7690	0.3076	15	0.7680	0.3072	12	0.7420	0.2968
12	0.7305	0.2922	16	0.7350	0.2940	13	0.6650	0.2660
13	0.6920	0.2768	17	0.7020	0.2808	14	0.6140	0.2456
14	0.6535	0.2614	18	0.6690	0.2676	15	0.4860	0.1944
15	0.5765	0.2306	19	0.5020	0.2008	16	0.4600	0.1840
16	0.4995	0.1998	21	0.4690	0.1876	17	0.4090	0.1636
17	0.4225	0.1690	22	0.3690	0.1476	18	0.3320	0.1328
18	0.3455	0.1382	23	0.1690	0.0676	19	0.2550	0.1020
19	0.3070	0.1228	24	0.1020	0.0408	20	0.1780	0.0712
20	0.2300	0.0920	28	0.0350	0.0140	21	0.1270	0.0508
21	0.1915	0.0766				22	0.0240	0.0096
22	0.1530	0.0612						
23	0.1145	0.0458						
26	0.0760	0.0304						
28	0.0375	0.0150						

Data Sources:

Tyrannosaurus rex and *Gorgosaurus libratus*: Erickson, G. M., *et al.* ***Science*** 313, 213–217 (2006).
Albertosaurus sarcophagus: Erickson, G. M., *et al.* ***Can. J. Earth Sci.*** 47, 1269–1275 (2010).

Figure S1

The characteristic life, α, is measured by detecting the interception point between s(x) and s(α) = exp(−1). The α estimate is obtained from a linear regression from two data points that exist just above (+1p) and just below (-1p) the alpha point.

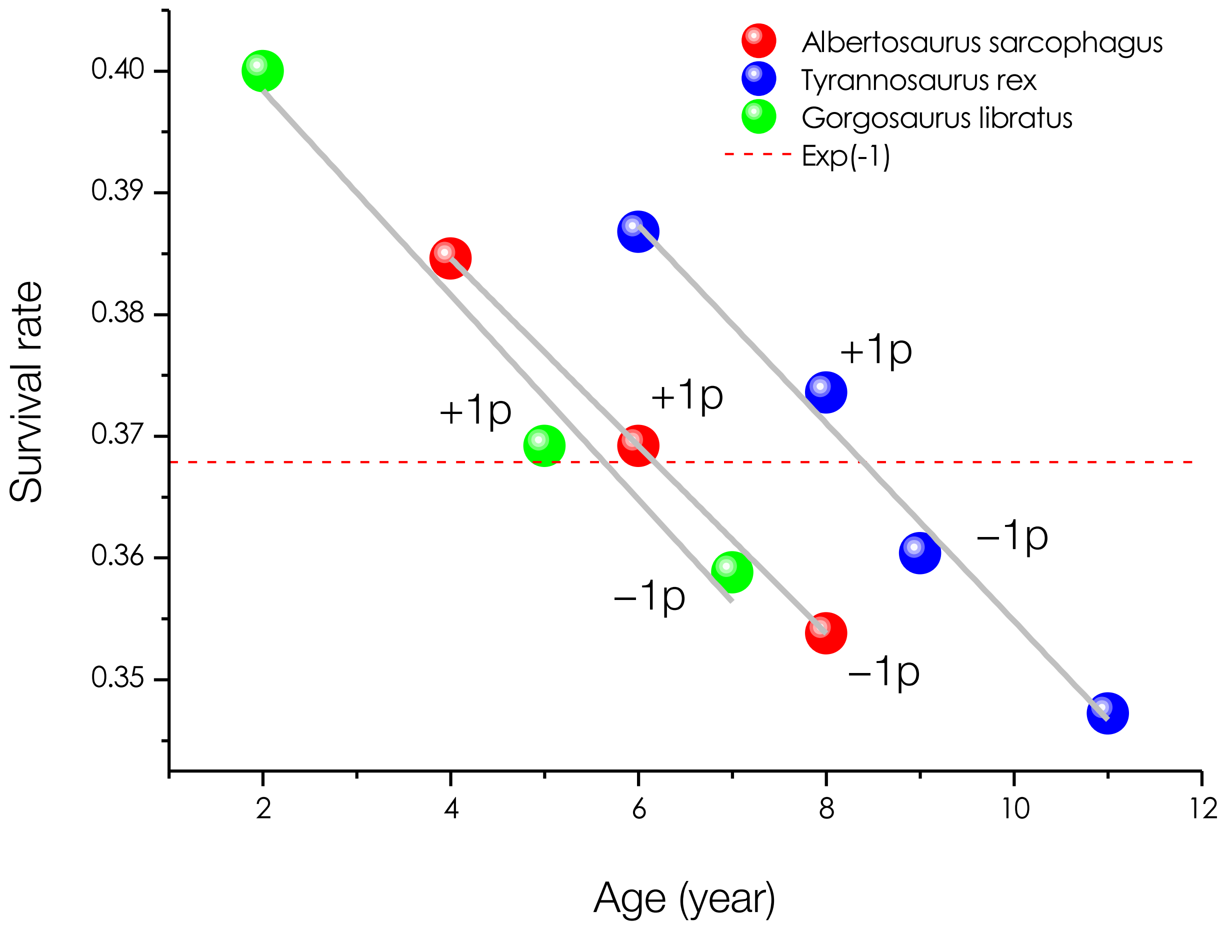

Supplement: Supplementary Information [file srep19554-s1.pdf]
